# Determinants of quality of life decrease in family caregivers of care-dependent patients: a longitudinal study

**DOI:** 10.1007/s11136-024-03814-w

**Published:** 2024-10-19

**Authors:** Marcus Luciano de Oliveira Tavares, Adriano Marçal Pimenta, Cristina García-Vivar, Mark Anthony Beinner, Lívia Cozer Montenegro

**Affiliations:** 1 Federal University of Minas Gerais, Belo Horizonte, MG Brazil; 2 Federal University of Paraná, Curitiba, PR Brazil; 3 Navarra Institute for Health Research, Public University of Navarre, Calle Irunlarrea, Pamplona, 31008 Navarra Spain

**Keywords:** Caregivers, Quality of life, Family, Family Nursing, Public Policy

## Abstract

**Purpose:**

Family caregivers of care-dependent patients experience a decline in their Quality of Life (QoL). However, the determinants contributing to this decrease in QoL are still not fully understood. Therefore, this study aimed to estimate prospectively the determinants contributing to decreased QoL among family caregivers of care-dependent patients.

**Methods:**

This longitudinal study involved 135 family caregivers in Brazil. Data were collected at baseline from October 2016 to August 2017, and at follow-up from December 2021 to July 2022. During both periods, we administered a questionnaire that covered sociodemographic, health, and lifestyle characteristics of the participants; the Barthel Index to assess the dependency level of the patients; and the WHOQOL-bref to assess the caregivers’ QoL.

**Results:**

Both caregivers and care-dependent patients were more frequently elderly (44.4% *versus* 74.6%), female (79.3% *versus* 61.5%), and had non-communicable disease (60.0% *versus* 94.3%) at baseline. Most patients experienced a worsening in their level of dependency (59.8%), while over a third of family caregivers (34.8%) reported a decline in their General Quality of Life Index. Eight determinants of decreased QoL were identified: four protective factors (religious faith, physical activity, sharing caregiving responsibilities, and sufficient sleep) and four risk factors (patient hospitalization in the past year, patient increased care dependency, older family caregiver age, and longer caregiving duration).

**Conclusion:**

Many factors influencing caregiver QoL are modifiable through intervention, underscoring the need for public policies to support family caregivers. Healthcare professionals can play a vital role in promoting protective factors and addressing risk factors to enhance caregiver QoL.

## Introduction

The shifts in demographics and epidemiological patterns have highlighted a rise in the elderly population, alongside an escalation in risk factors associated with Non-Communicable Diseases (NCDs). This, in turn, could lead to a surge in the prevalence of NCD-related complications and debilitating injuries, rendering a significant portion of the population reliant on caregiving [1, 2].

Illness, and consequently, the ensuing dependency on care, ripples through the lives of all family members, with a pronounced impact on family caregivers [3, 4]. Extensive research has focused on family caregivers of individuals with chronic illnesses, given their pivotal role in providing support to care-dependent patients and upholding long-term care systems across various nations [5–7].

Empirical evidence has demonstrated a significant impact on the quality of life (QoL) of family caregivers, stemming from the substantial burden of long-term care duties and their unwavering commitment to caring for their loved ones [5, 6].

While numerous studies delve into the repercussions of caregiving on the QoL of family caregivers, most of them employ cross-sectional methodologies, thereby constraining the ability to establish causal relationships [8]. Consequently, the need to conduct longitudinal studies arises, as they are essential for elucidating the factors that either diminish or elevate the QoL of family caregivers over time. Insights gleaned from longitudinal investigations can effectively guide policymakers and healthcare practitioners in formulating interventions aimed at ameliorating the negative effects of care dependency on the QoL of family caregivers.

Thus, this study aimed to estimate prospectively the determinants contributing to decreased QoL among family caregivers of care-dependent patients.

## Methods

### Design

This was an epidemiological, observational, longitudinal, and analytical study, coupled to a broader mixed-methods study that investigated the factors associated with changes in the QoL of family caregivers of care-dependent patients.

Baseline data were collected between October 2016 and August 2017, while follow-up data were gathered between December 2021 and July 2022.

Due to the study design, the tool Strengthening the Reporting of Observational Studies in Epidemiology (STROBE) [9] was used to provide structure and guidance to the presentation of findings in this paper.

### Setting

The study was conducted in two Primary Health Care (PHC) facilities, located in the city of Belo Horizonte, Minas Gerais State, Brazil.

### Population and sample

The selection of baseline participants for this study followed a meticulous process, beginning with the crucial step of establishing the term “family caregiver” as an individual who offers care to a family member without any form of financial remuneration. To identify eligible participants for this study, an exhaustive examination of all care-dependent patients within the study region was undertaken. Subsequently, care-dependent patients and their respective family caregivers were invited to participate.

The inclusion criteria encompassed care-dependent patients and unpaid family caregivers aged 18 years or older. Family caregivers whose care-dependent patients were categorized as “independent” following the assessment of dependency levels using the Barthel Index [10] were excluded from the study.

Because this was a longitudinal study, losses were expected. Cases of death or change of family caregiver were considered losses, in addition to refusal to participate. To address these scenarios, the following strategies were implemented: (1) In the case of a patient’s death, endeavors were made to establish communication with the family caregiver. If their willingness to partake in the study was confirmed, their participation was considered valid up to the date of the patient’s death; (2) Family caregivers whose care-dependent patients underwent a change in residence were still retained within the study cohort.

The process of corroborating this information was carried out during a meeting with community health workers – individuals who bridge the connection between the PHC facility and the community and maintain communication with the family caregivers previously engaged in the baseline study.

Among the initial 139 family caregivers interviewed at baseline [2], four individuals withdrew from the study – two due to a decision to discontinue participation and two who unfortunately passed away. As a result, the ultimate study cohort encompassed 135 care-dependent patients and their corresponding family caregivers.

### Variables and measurement instruments

At both baseline and follow-up, we applied the same measurement instruments: (1) A questionnaire developed by the research team to assess the sociodemographic, health status, and lifestyle characteristics of family caregivers and their care-dependents; (2) The Barthel Index [10], a widely recognized tool for assessing an individual’s ability to perform daily activities, was used to assess the level of patients care-dependency; (3) The World Health Organization Quality of Life – Bref (WHOQOL–bref), which was linguistically translated and validated for Brazilian Portuguese [11]. The WHOQOL-bref consists of 26 questions: two general inquiries about overall quality of life and 24 questions addressing specific domains of the original instrument. The resulting score ranges from 0 to 100, with higher scores indicating better quality of life.

### Data collection

Data collection at both baseline and follow-up was conducted through interviews carried out by a pair of trained researchers: a nurse who was a doctorate fellow, and an undergraduate nursing student. These interviews took place in a quiet room within the patients’ home, ensuring an environment devoid of disturbances and allowing family caregivers the privacy needed to respond to the questions. The period between the baseline and follow-up stages spanned roughly five years, facilitating a more comprehensive evaluation of the observed changes in the QoL experienced by the family caregivers.

### Data analysis

The baseline and follow-up databases were meticulously constructed utilizing the EpiInfo 7^®^ software. To ensure precision and minimize inconsistencies, the data was entered by two researchers actively involved in the data collection process. After data entry and a thorough check for any inconsistencies, the files were converted for analysis using the Stata 13^®^ software. A unique identification number (ID) was assigned to each participant, which remained consistent across both the baseline and follow-up databases.

In this study, our focus revolved around examining changes in quality of life (QoL) over time, serving as the outcome variable. To achieve this, we computed the deltas of QoL modifications (GQOLI and domains) by subtracting the scores obtained at the follow-up assessment from those collected at baseline. These score differences were subsequently categorized into three distinct groups: maintained (follow-up scores – baseline scores = zero), decreased (follow-up scores – baseline scores < zero), and increased (follow-up scores – baseline scores > zero). For the multivariable data analysis, the maintained and increased categories were grouped. Consequently, the outcome variable was binary: maintained/increased (serving as the reference category) and decreased.

Initially, we performed a characterization of care-dependent patients, and their family caregivers based on several aspects, presenting absolute and relative frequencies, means and standard deviations of sociodemographic, health status and lifestyle variables, degree of dependency and QoL.

To estimate the determinants of a decrease in the family caregiver’s QoL, a hierarchical multivariable analysis using the Poisson regression was conducted. For this study, for the multivariable analysis, the variables were divided into two blocks, following a theoretical model based on the findings of a prior study conducted by [2]. Notably, the mentioned study suggested that the attributes of the family caregivers themselves exerted a stronger association with their QoL than the characteristics of their care-dependent recipients. Consequently, in the distal block, we encompassed the variables concerning the care-dependent patient, while in the proximal block, we focused on the variables related to the family caregiver.

Variables associated with decreasing in the family caregiver’s QoL at a statistical significance level of 20% during the bivariable analysis were selected for the final model. These variables from the distal block were added to the final model in descending order of statistical significance and sequentially removed using the backward method until only those with a significance level below 5% remained. This process was repeated for the variables in the subsequent blocks. As a result, variables from earlier blocks were used to adjust those in the later blocks.

Furthermore, we conducted a multivariable sensitivity analysis, excluding 13 participants whose care-dependent patients passed away during the follow-up period.

### Ethical considerations

This study was conducted in accordance with the principles outlined in the Declaration of Helsinki, and all procedures involving study participants were approved by the Research Ethics Committee of Federal University of Minas Gerais (Brazil) under Registration No. 47529021.6.0000.5149. Moreover, informed consent was obtained from all participants.

## Results

The care-dependent patients were predominantly female (61.5%) and elderly (74.6%), with elementary school (59.0%) and a median family income of R$ 2,500 (US$ 525) at baseline. The family caregivers were more likely to be female (79.3%), adults (55.6), married (52.6%) and with elementary school (57.8%) at baseline (Table 1).

**Table 1:** Baseline and follow-up characteristics of dependents and their family caregivers. Belo Horizonte, MG, Brazil, 2016–2022

Characteristics	Dependent (*n* = 122)				Caregiver (*n* = 135)			
	*n* or Median	% or Interquartile range	*n* or Median	% or Interquartile range	*n* or Median	% or Interquartile range	*n* or Median	% or Interquartile range
Gender								
Female	75	61.5	75	61.5	107	79.3	107	79.3
Male	47	38.5	47	38.5	28	20.7	28	20.7
Age (*years*)^§^								
Adult (*18–59*)	31	25.4	29	23.8	75	55.6	59	44.0
Elderly (*≥ 60*)	91	74.6	93	76.2	60	44.4	75	56.0
Marital status								
Single/Divorced	44	36.1	43	35.3	49	36.3	50	37.0
Married	40	32.7	40	32.7	71	52.6	70	51.9
Widow	38	31.2	39	32.0	15	11.1	15	11.1
Children								
No	36	29.5	36	29.5	36	26.7	36	26.7
Yes	86	70.5	86	70.5	99	73.3	99	73.3
Education								
Iliterate	36	29.5	36	29.5	3	2.2	3	2.2
Elementary school	72	59.0	72	59.0	78	57.8	76	56.3
High school or college	14	11.5	14	11.5	54	40.0	56	41.5
Income (*R$*)^*;**^	*2*, *500*^*a*^	*1*, *874-3*, *300*^*a*^	*3*, *636*^*a*^	*2*, *414-4*, *848*^*a*^	*937* ^b^	*0–1*, *000*^b^	*1*, *212*^b^	*0–1*, *424*^b^
Physical activity (*min/wk*)^§^								
0	-	-	-	-	91	67.4	102	75.6
1 to 149	-	-	-	-	19	14.1	15	11.1
≥ 150	-	-	-	-	25	18.5	18	13.3
Leasure activity								
No	-	-	-	-	60	44.4	62	45.9
Yes	-	-	-	-	75	55.6	73	54.1
Religious faith								
No	-	-	-	-	21	15.6	25	18.5
Yes	-	-	-	-	114	84.4	110	81.5
Sleep (*hours/night*)^*^	*9*	*8–10*	*10*	*8–10*	*7*	*6–8*	*8*	*6–8*
NCD^†;§^								
No	7	5.8	0	0.0	54	40.0	26	19.3
Yes	115	94.3	122	100.0	81	60.0	109	80.7
Dependency time (*years*)^*^	*5*	*2–15*	*10.1*	*7.1–19.9*	-	-	-	-
Hospitalized in the past 12 months^†^								
No	74	60.7	110	90.2	-	-	-	-
Yes	48	39.3	12	9.8	-	-	-	-
Time as a caregiver (*years*)^**^	-	-	-	-	*5*	*2–12*	*10.1*	*6.9–15.3*
Number of dependents^§^								
1	-	-	-	-	116	85.9	131	97.0
2 or more	-	-	-	-	19	14.1	4	3.0
Sharing caregiver responsibilities								
No	-	-	-	-	71	52.6	81	60.0
Yes	-	-	-	-	64	47.4	54	40.0
Caregiver training								
No	-	-	-	-	120	88.9	112	83.0
Yes	-	-	-	-	15	11.1	23	17.0

Note. NCD = Noncommunicable disease; ^a^Family income; ^b^Individual income; ^*^p-value from Wilcoxon test < 0.05 for differences in dependents’ characteristics; ^**^p-value from Wilcoxon test < 0.05 for differences in caregivers’ characteristics; ^†^*p*-value from proportion comparison test < 0.05 for differences in dependents’ characteristics; ^§^*p*-value from proportion comparison test < 0.05 for differences in caregivers’ characteristics

Between baseline and follow-up, some characteristics of dependents had statistically significant changes (*p* < 0.05), such as income (increased), hours of sleep (increased), and frequencies of NCD (increased) and hospitalization in the past 12 months (decreased). Among the caregivers, they also had statistically significant changes (*p* < 0.05) in some of their characteristics, such as age (increased number of elderly), income (increased), physical activity (increased number of sedentary), and frequencies of NCD (increased) and two or more dependents under their care (decreased) (Table 1).

With regards to the level of patient care dependency, an analysis of the variation between baseline and follow-up revealed noteworthy differences. A continuous analysis of this variable indicated a mean reduction of -11.2 (standard deviation - SD = 13.8), signifying a deterioration in the degree of care dependency over the observed period. In a categorical perspective, encompassing four strata, a significant shift was observed among individuals initially categorized as slightly dependent (Table 2).

**Table 2:** Variation of dependents’ level of dependency between baseline and follow-up (*n* = 122). Belo Horizonte, MG, Brazil, 2016–2022

Degree of dependency	Mean	SD	Median	Interquartile Range
Barthel Index (*continuous*)				
Follow-up	41.4	26.5	47.5	20.0–60.0
Baseline	52.5	30.5	57.5	25.0–80.0
Modification (*general difference*)^a^	-11.2	13.8	-10.0	-15.0–0.0
Worsering (*n* = 73; 59.8%)	-18.5	13.4	-15.0	-20.0 – -10.0
Stable (*n* = 49; 40.2%)	-	-	-	-
Barthel Index (*categorical*)				
Follow-up [*n*, % (CI 95%)]				
Total	33		27.1% (19.8 – 35.7%)	
Severe	16		13.1% (8.1 – 20.4%)	
Moderate	28		22.9% (16.2 – 31.3%)	
Slight^b^	45		36.9% (28.6 – 45.9%)	
Barthel Index (*categorical*)				
Baseline [*n*, % (CI 95%)]				
Total	26		21.3% (14.8 – 29.5%)	
Severe	14		11.5% (6.8 – 18.5%)	
Moderate	21		17.2% (11.4 – 25.0%)	
Slight^b^	61		50.0% (41.0 – 58.9%)	

Note. SD = Standard Deviation; CI = Confidence Intervals; ^a^Statistically significant differences between baseline and follow-up Quality of Life scores according to the Wilcoxon test (*p* < 0.05); ^b^Statistically significant differences, as there was no intersection between the 95% Confidence Intervals (95% CI) of the follow-up and baseline proportions

Upon comparing the QOL of family caregivers at baseline and follow-up, a significant decline in levels was evident across all four QOL domains as well as the general index. Notably, the most substantial reduction was observed in the General Quality of Life Index (GQOLI), displaying a mean loss of -5.9 (SD = 16.0) (Table 3).

**Table 3:** Variation of family caregivers’ quality of life at baseline and follow-up (*n* = 135). Belo Horizonte, MG, Brazil, 2016–2022

Quality of life	Mean	SD	Median	Interquartile Range
GQOLI				
Follow-up	58.9	23.7	62.5	37.5–75.0
Baseline	64.9	20.4	62.5	50.0–75.0
Modification (*general difference*)^a^	-5.9	16.0	0.0	-12.5–0.0
Decreased (*n* = 47; 34.8%)	-22.3	12.7	-12.5	-25.0 – -12.5
Stable (*n* = 77; 57.0%)	-	-	-	-
Increased (*n* = 11; 8.2%)	22.7	15.6	12.5	12.5–25.0
Physical domain				
Follow-up	69.1	18.3	75.0	57.1–82.1
Baseline	70.7	18.0	75.0	57.1–82.1
Modification (*general difference*)^a^	-1.6	8.6	0.0	-3.5–0.0
Decreased (*n* = 45; 33.3%)	-9.5	7.5	-7.1	-14.2 – -3.5
Stable (*n* = 71; 52.6%)	-	-	-	-
Increased (*n* = 19; 14.1%)	10.9	9.1	7.1	3.5–14.2
Psychological domain				
Follow-up	65.1	18.7	70.8	50.0–79.2
Baseline	68.3	16.1	70.8	58.3–79.1
Modification (*general difference*)^a^	-3.3	8.1	0.0	-8.3–0.0
Decreased (*n* = 51; 37.8%)	-11.1	7.2	-8.3	-16.6 – -4.1
Stable (*n* = 68; 50.4%)	-	-	-	-
Increased (*n* = 16; 11.8%)	7.2	5.5	4.1	4.1–8.3
Social domain				
Follow-up	66.5	21.8	66.6	50.0–83.3
Baseline	69.8	20.9	75.0	58.3 – 83.3
Modification (*general difference*)^a^	-3.2	9.9	0.0	0.0–0.0
Decreased (*n* = 28; 20.7%)	-18.7	10.7	-16.6	-25.0 – -8.3
Stable (*n* = 101; 74.8%)	-	-	-	-
Increased (*n* = 6; 4.5%)	13.8	10.0	8.3	8.3–16.6
Environmental domain				
Follow-up	61.2	14.5	62.5	53.1–68.7
Baseline	62.5	13.6	62.5	53.1–71.8
Modification (*general differencel*)^a^	-1.3	4.8	0.0	-3.1–0.0
Decreased (*n* = 39; 28.9%)	-7.1	4.0	-6.2	-9.3 – -3.1
Stable (*n* = 79; 58.5%)	-	-	-	-
Increased (*n* = 17; 12.6%)	5.8	3.6	6.2	3.1–6.2

Note. SD = Standard Deviation; GQOLI = General Quality of Life Index; ^a^Statistically significant differences between baseline and follow-up Quality of Life scores according to the Wilcoxon test (*p* < 0.05)

Table 4 illustrates the factors independently associated with the decreasing QOL of family caregivers. In the context of GQOLI, a decrease in the QOL of family caregivers was associated with certain factors. Notably, a care-dependent patient’s hospitalization within the last 12 months was linked to a higher risk (Relative Risk – RR = 1.62; 95% Confidence Interval – 95% CI = 1.05–2.49), alongside a deterioration in the care-dependent patient’s level of dependency (RR = 1.90; 95% CI = 1.13–3.18). Additionally, advancing age of the family caregiver was identified as a contributing risk factor (RR = 2.19; 95% CI = 1.41–3.42). Conversely, protective factors encompassed having a religious faith (RR = 0.35; 95% CI = 0.23–0.55) and jointly sharing caregiver responsibilities (RR = 0.62; 95% CI = 0.41–0.94).

**Table 4:** Factors independently associated with changes in the quality of life of family caregivers. Belo Horizonte, MG, Brazil, 2016–2022

Characteristics	Domains									
	GQOLI (*n* = 122)^a^		Physical (*n* = 135)		Pyschological (*n* = 135)		Social (*n* = 135)		Environmental (*n* = 135)	
	RR	CI 95%	RR	CI 95%	RR	CI 95%	RR	CI 95%	RR	CI 95%
**Dependents**										
Hospitalized (*last 12 months*)										
No	1	Ref.	-	-	-	-	-	-	-	-
Yes	**1.62**	**1.05–2.49**	-	-	-	-	-	-	-	-
Worsening degree of dependence										
No	1	Ref.	-	-	-	-	-	-	-	-
Yes	**1.90**	**1.13–3.18**	-	-	-	-	-	-	-	-
**Caregivers**										
Age (*years*)										
Adult (*18–59*)	1	Ref.	-	-	-	-	-	-	-	-
Elderly *(≥ 60*)	**2.19**	**1.41–3.42**	-	-	-	-	-	-	-	-
Physical activity (*min/wk*)										
0	-	-	-	-	1	Ref.	-	-	-	-
1 to 149	-	-	-	-	0.60	0.29–1.29	-	-	-	-
≥ 150	-	-	-	-	**0.38**	**0.15–0.95**	-	-	-	-
Religious faith			-							
No	1	Ref.	1	Ref.	-	-	1	Ref.	1	Ref.
Yes	**0.35**	**0.23–0.55**	**0.53**	**0.33–0.85**	-	-	**0.32**	**0.16–0.66**	**0.52**	**0.30–0.90**
Sleep (*hours/night*)	-	-	-	-	-	-	**0.83**	**0.72–0.96**	-	-
Time as a caregiver (*years*)	-	-	**1.02**	**1.01–1.04**	-	-	-	-	**1.02**	**1.01–1.03**
Share caregiving responsibilities										
No	1	Ref.	1	Ref.	1	Ref.	1	Ref.	1	Ref.
Yes	**0.62**	**0.41–0.94**	**0.39**	**0.22–0.67**	**0.55**	**0.34–0.89**	**0.39**	**0.19–0.80**	**0.42**	**0.22–0.77**

Note. GQOLI = General Quality of Life Index; RR = Relative Risk; ^a^For GQOLI analysis, caregivers, whose dependents died during the follow-up period, were excluded

Concerning the physical domain, the QOL among family caregivers showed a decline correlated with the number of years they had spent in their caregiving role (RR = 1.02; 95% CI = 1.01–1.04). On the other hand, certain protective factors were identified against a decrease in QOL within the Physical domain. Specifically, maintaining religious faith was associated with a reduced risk (RR = 0.53; 95% CI = 0.33–0.85), as well as sharing caregiver responsibilities (RR = 0.39; 95% CI = 0.22–0.67), as detailed in Table 4.

Furthermore, our analysis revealed notable findings in the psychological domain. Engaging in physical activity for more than 150 min per week was associated with a lowered risk of QOL decrease (RR = 0.38; 95% CI = 0.16–0.93), as well as sharing caregiver responsibilities (RR = 0.55; 95% CI = 0.34–0.89), offering protection against a decline in QOL (Table 4).

Moreover, our analysis revealed noteworthy results in the social relations domain. Religious faith (RR = 0.32; 95% CI = 0.16–0.66), along with longer hours of sleep per day (RR = 0.83; 95% CI = 0.72–0.96) and sharing caregiver responsibilities (RR = 0.38; 95% CI = 0.18–0.78) emerged as protective factors against a decrease in QOL (Table 4).

In examining the environmental domain, the number of years spent as a caregiver (RR = 1.02; 95% CI = 1.01–1.03) was identified as a risk factor for a decrease in QOL. Conversely, religious faith (RR = 0.52; 95% CI = 0.30–0.90) and sharing caregiver responsibilities (RR = 0.44; 95% CI = 0.22–0.77) emerged as protective factors against such a decrease in this domain (Table 4).

Lastly, we conducted a multivariate sensitivity analysis, excluding 13 participants whose care-dependent loved ones had passed away during the follow-up period. The results were largely consistent with those presented earlier, except for the environmental domain. Specifically, in this domain, the variables of religious faith and years as a caregiver lost their statistical significance (Table 5).

**Table 5:** Sensitivity analysis of factors independently associated with changes in the quality of life of family caregivers. Belo Horizonte, MG, Brazil, 2016–2022 (*n* = 122)

Characteristics	Domains									
	GQOLI^a^		Physical		Psychological		Social		Environmental	
	RR	CI 95%	RR	CI 95%	RR	CI 95%	RR	CI 95%	RR	CI 95%
**Dependents**										
Hospitalized (*last 12 months*)										
No	1	Ref.	-	-	-	-	-	-	-	-
Yes	**1.62**	**1.05–2.49**	-	-	-	-	-	-	-	-
Worsening degree of dependence										
No	1	Ref.	-	-	-	-	-	-	-	-
Yes	**1.90**	**1.13–3.18**	-	-	-	-	-	-	-	-
**Caregivers**										
Age (*years*)										
Adult (*18–59*)	1	Ref.	-	-	-	-	-	-	-	-
Elderly (*≥ 60*)	**2.19**	**1.41–3.42**	-	-	-	-	-	-	-	-
Physical activity (*min/sem*)										
0	-	-	-	-	1	Ref.	-	-	-	-
1 to 149	-	-	-	-	0.56	0.28–1.15	-	-	-	-
≥ 150	-	-	-	-	**0.39**	**0.16–0.95**	-	-	-	-
Religious faith			-							
No	1	Ref.	1	Ref.	-	-	1	Ref.	-	-
Yes	**0.35**	**0.23–0.55**	**0.60**	**0.37–0.97**	-	-	**0.48**	**0.25–0.92**	**-**	**-**
Sleep (*hours/night*)	-	-	-	-	-	-	**0.84**	**0.73–0.97**	-	-
Time as a caregiver (*years*)	-	-	**1.02**	**1.01–1.04**	-	-	-	-	**-**	**-**
Share caregiving responsibilities										
No	1	Ref.	1	Ref.	1	Ref.	1	Ref.	1	Ref.
Yes	**0.62**	**0.41–0.94**	**0.40**	**0.23–0.69**	**0.56**	**0.35–0.89**	**0.40**	**0.20–0.81**	**0.45**	**0.25–0.82**

Note. GQOLI = General Quality of Life Index; RR = Relative Risk; ^a^For GQOLI analysis, caregivers, whose dependents died during the follow-up period, were excluded

## Discussion

This study analyzed the determinants impacting the decrease in QOL among family caregivers of care-dependent patients over five years. We identified eight determinants: four protective factors (religious faith, sharing caregiver responsibilities, physical activity, and sufficient sleep) and four risk factors (patient hospitalization in the past year, increased care dependency, older caregiver age, and longer caregiving duration).

### Protective factors that contribute to the quality of life of family caregivers

#### Sharing caregiver responsibilities

In this study, sharing caregiver responsibilities emerged as a key protective factor, positively influencing all domains of family caregiver QOL. Consequently, healthcare practitioners should acknowledge its importance, as it helps alleviate caregiving burdens [12]. McCauley et al. [13] found that assistance from other family members enhances emotional engagement between patients and family caregivers in palliative care, mitigating caregiver suffering. However, “the perceived lack of support from family caregivers on the part of other family members can make it more difficult for the former to adjust to the demands of care” (13:883). Family Systems Theory posits that flexible hierarchies and roles influence family dynamics [14, 15]. Sharing family caregiving responsibilities can alleviate the caregivers´ burden, improve their quality of life, and promote the overall equilibrium of family functioning [14]. Family caregivers are often seen as part of home health teams [16]. Recognizing that the family is greater than the sum of its parts [17], helps healthcare professionals understand family dynamics, identify vulnerabilities and strengths, and develop tailored care interventions. This approach distributes caregiving duties, easing the caregiving routine and reducing the caregiver’s burden, thereby safeguarding their well-being [12, 18, 19]. Incorporating paid caregivers is crucial for sharing care responsibilities, providing functional and emotional support to care-dependent individuals and family caregivers.

However, many families cannot afford this support, highlighting the need for government efforts to integrate professional caregivers into the healthcare system. This could be achieved through Primary Health Care (PHC) services or other initiatives, alleviating the burden on family caregivers [20]. Encouraging families to see the benefits of shared caregiving can significantly improve caregivers’ quality of life. Family nurses in PHC can lead this effort, even if it involves reshaping family dynamics and rules. This approach, supported by experts like Shajani and Snell [15], can transform caregiving dynamics for the better.

#### Religious faith

Our findings highlight religious faith as a protective factor for family caregivers’ quality of life across the GQOLI and in physical and social domains. This aligns with literature linking faith and spirituality to higher quality of life [21]. Religion provides crucial spiritual support often overlooked by healthcare practitioners, as noted by patients and caregivers worldwide [22]. Active involvement in religious practices positively affects physical health and overall well-being, reducing the impact of illnesses [23]. Religious coping, widely used by caregivers -especially older individuals, those with increased caregiving responsibilities, spouses or mothers, and those caring for chronically ill or dependent patients utilizes religion as a coping mechanism [24]. Additionally, social support within religious communities enhances quality of life [25].

#### Physical activity

In this study, engaging in physical activity emerged as another pivotal protective factor for family caregiver’s QOL, impacting not only physical well-being but also enhancing mental and social dimensions [26]. Physical activity improves cardiovascular fitness, muscular strength, bone health, and metabolic function, and also positively affects mental and social aspects [26].

Physical activity strongly correlates with improved quality of life, supported by extensive research [27]. The World Health Organization recommends 150 to 300 min of moderate-intensity physical activity weekly for adults [28]. Family caregivers also benefit from physical activity, enhancing their psychosocial and physical health, and involving their care-dependent member during the activity can further improve family health [12].

#### Sufficient sleep

Our study revealed a significant association between longer sleep and improved QOL for caregivers, highlighting sleep’s critical role in safeguarding QOL. Importantly, insufficient sleep quality, often due to inadequate rest, is linked to lower QOL [29].

Further supporting this insight is the recognition that family caregivers often experience increased insomnia, susceptibility to sleep apnea, poor sleep quality, and compromised QOL [30]. These findings highlight the complex relationship between sleep and QOL, underscoring the need to address sleep-related issues to enhance caregivers’ overall well-being and QOL.

### Negative determinants impacting the quality of life of family caregivers

#### Increased care dependency

Our findings strongly indicate that higher levels of care dependency in the family markedly reduce the caregiver’s QOL, especially concerning their General Quality of Life Index (GQOLI). Caring for highly dependent individuals complicates the task, requiring increased dedication and effort from the family caregiver. Often, they assume this role without sufficient guidance or support from other family members or healthcare professionals, resulting in significant stress and strain [31, 32].

#### Older caregiver age

Aging encompasses various factors that affect an individual’s physical state, including physiological changes and psychosocial dynamics. These factors interact to determine an individual’s intrinsic capacity, which includes both physical and mental abilities [33]. In our study, we found that older age among family caregivers is associated with a decline in their quality of life measured by the GQOLI. This could be explained by the significant burden of caring for a dependent individual. The demands of caregiving strain the caregiver’s physical, mental, and cognitive capacities, which are naturally affected by aging and the challenges of caregiving [3, 4, 8].

In this context, a clear cycle of deterioration emerges, deepening aspects of the caregiver’s aging trajectory. Aging naturally involves a decline in intrinsic capabilities [33], and when combined with caregiving demands [3, 8], it can result in aging individuals coping with compromised health. Paradoxically, this can accelerate the erosion of their intrinsic capabilities, perpetuating this distressing cycle.

#### Care-dependent patient hospitalization in the past year

Our study found that family caregivers whose care-dependent members were hospitalized in the past year faced an increased risk of declining QOL. This heightened risk is due to the considerable strain caused by the hospitalization process on caregivers, as well as the significant efforts required for the family to regain functional equilibrium afterward.

Hospitalization necessitates major adjustments, characterized by feelings of fear, insecurity, and anxiety. This period can be viewed as a potentially traumatic event that disrupts family dynamics and destabilizes the entire household. For family caregivers, this experience is even more intense due to their direct involvement in complex and sensitive situations within medical settings [34]. To mitigate the impact of hospitalization, it becomes imperative for the family to establish a rotating caregiving schedule, alleviating the burden on the caregiver. This approach also fosters a broader support network including friends, neighbors, associations, and other family members, enhancing overall support [34].

#### Longer caregiving duration

Caring for a chronically dependent individual is long-term, often because the condition tends to be irreversible. Our study found that over time, dependency levels either worsen or stabilize, with no family member showing improvement. Thus, the role of a family caregiver is continuous and influenced by various factors.

In our study, we assessed the persistent nature of caregiving by measuring the time family caregivers spent on their duties. This aspect emerged as a potential risk factor for reduced QOL, especially in the physical domain. This finding suggests that the decline in caregivers’ physical QOL may be linked to their worsening health and decreased intrinsic capacity associated with aging [33].

Caregiving demands physical effort from caregivers, which over time can lead to conditions such as musculoskeletal disorders and mental health challenges. These issues can significantly impact caregivers’ well-being and their ability to fulfill caregiving responsibilities effectively [3].

Most of the factors identified in our study are amenable to intervention, which can either mitigate or enhance their impact. These factors include sharing caregiver responsibilities, engaging in physical activity, ensuring sufficient sleep duration, addressing hospitalization history, managing care-dependency levels, and addressing caregiving duration. However, achieving these changes requires the establishment of collaborative intersectoral approaches that involve policymakers, administrators, healthcare practitioners, and families working together.

### Limitations of the study

Given the specific attributes of the studied region and the longitudinal nature of our research, there were drop-outs, which, while not statistically significant, could potentially impact our outcomes. The generalization of our findings beyond the studied population must be done with caution, and any comparisons with findings from other studies should be approached with sensitivity to the distinct characteristics of the assessment tools employed.

## Conclusion

Over the years, the demand for family caregivers is expected to rise globally, also in Brazil. Considering this, we conclude that the implementation of supportive policies for family caregivers and strategic interventions by healthcare professionals could play a pivotal role in enhancing the QOL for family caregivers of care-dependent patients. These efforts could manifest as either bolstering protective factors or mitigating risk factors. Notably, these initiatives would not only yield benefits for caregivers but could also lead to decreased healthcare expenditures by the state. This is particularly relevant as caregivers with diminished QOL are susceptible to health issues, potentially straining public health services.

Establishing a comprehensive care network that extends support to families is an imperative need. This approach acknowledges that tending to the family unit directly influences its dynamics and overall functioning. Such a network not only mitigates the risk of illness within the family but also safeguards their holistic well-being—ranging from physical and mental health to social and economic aspects.
